# Editorial: Predicting Chronological Age From Structural Neuroimaging: The Predictive Analytics Competition 2019

**DOI:** 10.3389/fpsyt.2021.710932

**Published:** 2021-08-05

**Authors:** Lukas Fisch, Ramona Leenings, Nils R. Winter, Udo Dannlowski, Christian Gaser, James H. Cole, Tim Hahn

**Affiliations:** ^1^Institute for Translational Psychiatry, University of Münster, Münster, Germany; ^2^Department of Psychiatry and Psychotherapy, Jena University Hospital, Jena, Germany; ^3^Department of Neurology, Jena University Hospital, Jena, Germany; ^4^Department of Neuroimaging, Institute of Psychiatry, Psychology and Neuroscience, King's College London, London, United Kingdom; ^5^Computational, Cognitive and Clinical Neuroimaging Laboratory, Department King's College, London, United Kingdom

**Keywords:** sMRI, neuroimaging, brain-age, brain health, biomarker

Though aging is ubiquitous, the rate at which age-associated biological changes in the brain occur differs substantially between individuals. Building on this, the so-called *brain-age paradigm* (1) aims to estimate a brain's biological age (2) and may serve as a cumulative marker of disease-risk, functional capacity and residual lifespan (3). In a typical brain-age study, a machine learning model is trained on neuroimaging data—usually whole-brain structural T_1_-weighted Magnetic Resonance Imaging (MRI) data—to predict chronological age. This trained model is then used to evaluate neuroimaging data from previously unseen individuals and evaluated based on the brain-age gap as defined by the difference between predicted and chronological age.

The Predictive Analytics Competition (PAC) 2019 aimed to bring together machine learning and neuroimaging experts to improve existing brain-age models. Based on a large structural Magnetic Resonance Imaging (sMRI) dataset of *N* = 3,307 healthy individuals (see Table 1) provided by the two organizing sites (University of Münster, King's College London), the PAC 2019 pursued two goals: first, participants aimed to minimize the mean difference between chronological and predicted age (i.e., the brain-age gap). The second objective was to minimize the brain-age gap while keeping the Spearman correlation between the brain-age gap and chronological age below *r* = 0.10 to avoid the commonly observed bias of age estimations toward the mean age of the training dataset, as discussed by Treder et al.. The PAC 2019 featured 274 participants in 79 teams from across the globe and resulted in a great variety of submitted machine learning models.

**Table 1:** Overview of sample distributions.

**Sample**	***N***	**N males**	**Age mean**	**Age Std**.	**Age Min**.	**Age Max**.
Train	2,640	1,237	35.88	16.21	17	90
Test	660	321	35.40	15.86	17	89

*Std, Standard Deviation; Min, Minimum; Max, Maximum; HC, Healthy Controls*.

In this special issue, 8 publications including the top performing models of the PAC 2019, are described in detail to provide insights into cutting-edge brain-age modeling and an outlook on future developments. The PAC 2019 winning team of Gong et al. used lightweight 3D Convolutional Neural Networks (CNNs) based on the Fully Convolutional Network (4) and the VGG net (5) combined with different preprocessing steps to form a multimodal ensemble which was pre-trained on *N* = 14,503 samples from UK Biobank. This approach reached the lowest mean absolute errors (MAE) of 2.90 years and 2.95 years for the first and second objective of the PAC, respectively. The third and fourth place finishing teams also used an ensemble of differently preprocessed data and 3D CNN architectures (see Couvy-Duchesne et al.; Kuo et al.), however using more layers in these CNNs reaching an MAE of 3.33 years.

Deep learning models performed better than classic machine learning algorithms such as Support Vector Machines, Relevance Vector Machines or Gaussian Process Regression; only one of which reached an MAE of 3.09 years being submitted but not published. Further differentiating within the group of deep learning models, CNNs using 3D instead of 2D kernels are the natural choice as they exploit features across all three spatial dimensions of imaging data. In contrast to the deep 2D CNN architectures which reach top performance in popular computer vision challenges such as ImageNet and used for slice-level modeling by Ballester et al., age prediction appears to benefit from shallower CNN architectures, as Gong et al., and Da Costa et al. demonstrate. This suggests that we have either not reached sample sizes at which deeper architectures provide advantages or age-related changes in brain morphology are simple compared to natural scenes and objects. As the performance increase of neural networks can only be achieved with ever larger samples sizes, more recent brain-age studies include 10,000 and more samples (6, 7)—a trend that will surely accelerate as recruitment for large-scale studies such as UK Biobank continues. Gong et al. winning PAC model was pre-trained on *N* = 14,503 samples, emphasizing the benefit of transferring learnt representations from one large sample to another smaller dataset. Finally, using different pre-processing pipelines, as done by the top PAC submissions, appears to be beneficial as it implements a form of data augmentation, a method established in the field of computer vision to increase the data variability and thus improve generalization to new datasets.

The superior performance of non-linear and deep learning approaches for brain-age prediction, demonstrated empirically by Lombardi et al., contrasts with the finding in Schulz et al. (8) that for many predictive tasks in neuroimaging—from the prediction of personality traits to mental disorders—simple linear models perform at least on par with non-linear and deep learning models. This discrepancy might be explained by (1) the increasing sample sizes available today which allow deep learning models to be better estimated and (2) low dimensional data does not necessarily benefit from deep models, as shown by Soch in this issue.

Going beyond model performance, “explainability” (i.e., understanding which features are relevant for brain-age predictions) is gaining increasing attention. For example, first advances in explainable machine learning in the neuroimaging context were possible due to the use of U-Net models (9), patch-based brain age estimation (10) and visual attention (11). These methods shed light on which brain areas are most important for brain-age prediction. In Bintsi et al. (10) the ventricles and hippocampus were highlighted. Partially confirming these findings, in Dinsdale et al. (12) the region around the ventricles also showed the largest difference between a group with low and a group with high brain-age predictions. Explainability methods might pose an elegant way of feeding back information to the neuroscience community to expand our knowledge of the biological mechanisms involved in the aging process itself.

Quantifying the uncertainty of brain-age predictions is also crucial for a broader adoption of the brain-age paradigm in clinical contexts. For example, the Monte-Carlo Dropout Composite-Quantile Regression (MCCQR) developed by Hahn et al. (13) showed that correcting brain-age predictions for aleatory and epistemic uncertainty can (1) protect against spurious results which are overlooked in classic brain-age modeling and (2) dramatically increase the power to detect associations between the brain-age gap and mental disorders. On top of quantifying uncertainty (13), suggests the median average error instead of the mean average error or mean squared error as a loss function. Using this loss function regularizes against outliers i.e., samples which deviate from the major aging trajectory in the dataset.

Finally, performing brain-age prediction on raw MRI data would not only dramatically reduce required computation times, but also increases the usability of the model, making it more accessible for clinicians. To this end (14), developed a shallow 3D ResNet architecture which—in combination with a second neural network for automatic skull stripping—provides state-of-the-art performance (MAE = 2.84) based on raw T_1_-weighted MRI data.

Conceptually, as proposed in Bashyam et al. (15), with increased performance of brain age models, the possibility of models learning to completely correct for altered brain aging (e.g., caused by disease or life style) by extrapolation from the training data, could counter the utility of the brain-age as a biomarker. While (16) showed that this effect has not yet been empirically observed, the argument points toward a conceptual issue which could arise if models improve so much that they implicitly correct brain age for diseases. While certainly true, this issue could prove to be invaluable as such models would—by definition—enable an accurate direct classification of patients. Following this logic, transfer learning could be leveraged to exploit this feature for difficult classification tasks (e.g., the differentiation of depressive and bipolar patients) by firstly pre-training these models to predict age and successively fine-tune the resulting model for classification tasks.

In summary, the field of brain-age modelling—as evidenced not least by the participants of the PAC 2019—has made great progress, both conceptually and methodologically in recent years. With such a vibrant community, we are excited to see future developments and look forward to the first clinical applications.

## Author Contributions

All authors listed have made a substantial, direct and intellectual contribution to the work, and approved it for publication.

## Conflict of Interest

The authors declare that the research was conducted in the absence of any commercial or financial relationships that could be construed as a potential conflict of interest.

## Publisher's Note

All claims expressed in this article are solely those of the authors and do not necessarily represent those of their affiliated organizations, or those of the publisher, the editors and the reviewers. Any product that may be evaluated in this article, or claim that may be made by its manufacturer, is not guaranteed or endorsed by the publisher.
